# Mallotucin D, a Clerodane Diterpenoid from *Croton crassifolius*, Suppresses HepG2 Cell Growth via Inducing Autophagic Cell Death and Pyroptosis

**DOI:** 10.3390/ijms232214217

**Published:** 2022-11-17

**Authors:** Xiaoyong Dai, Fen Sun, Kexin Deng, Gaoyang Lin, Wenjing Yin, Huaqing Chen, Dongye Yang, Kewei Liu, Yubo Zhang, Laiqiang Huang

**Affiliations:** 1 Precision Medicine and Healthcare Research Center, Center for Biotechnology and Biomedicine, Shenzhen Key Laboratory of Gene and Antibody Therapy, State Key Laboratory of Chemical Oncogenomics, State Key Laboratory of Health Sciences and Technology, Tsinghua-Berkeley Shenzhen Institute (TBSI), Institute of Biopharmaceutical and Health Engineering, Shenzhen International Graduate School, Tsinghua University, Shenzhen 518055, China; 2 School of Life Sciences, Tsinghua University, Beijing 100084, China; 3 Department of Chemistry, Tsinghua University, Beijing 100084, China; 4 Guangdong Clinical Translational Center for Targeted Drug, Department of Pharmacology, School of Medicine, Jinan University, Guangzhou 510632, China; 5 Division of Gastroenterology and Hepatology, The University of Hongkong-Shenzhen Hospital, Shenzhen 518055, China

**Keywords:** HepG2 cell, mallotucin D, ROS, mitophagy, pyroptosis, BNIP3

## Abstract

Hepatocellular carcinoma (HCC) is a major subtype of primary liver cancer with a high mortality rate. Pyroptosis and autophagy are crucial processes in the pathophysiology of HCC. Searching for efficient drugs targeting pyroptosis and autophagy with lower toxicity is useful for HCC treatment. Mallotucin D (MLD), a clerodane diterpenoid from *Croton crassifolius,* has not been previously reported for its anticancer effects in HCC. This study aims to evaluate the inhibitory effects of MLD in HCC and explore the underlying mechanism. We found that the cell proliferation, DNA synthesis, and colony formation of HepG2 cells and the angiogenesis of HUVECs were all greatly inhibited by MLD. MLD caused mitochondrial damage and decreased the TOM20 expression and mitochondrial membrane potential, inducing ROS overproduction. Moreover, MLD promoted the cytochrome C from mitochondria into cytoplasm, leading to cleavage of caspase-9 and caspase-3 inducing GSDMD-related pyroptosis. In addition, we revealed that MLD activated mitophagy by inhibiting the PI3K/AKT/mTOR pathway. Using the ROS-scavenging reagent NAC, the activation effects of MLD on pyroptosis- and autophagy-related pathways were all inhibited. In the HepG2 xenograft model, MLD effectively inhibited tumor growth without detectable toxicities in normal tissue. In conclusion, MLD could be developed as a candidate drug for HCC treatment by inducing mitophagy and pyroptosis via promoting mitochondrial-related ROS production.

## 1. Introduction

Hepatocellular carcinoma (HCC) is the most common primary liver cancer with high malignancy and poor prognosis [1]. Because of chronic hepatitis infection, the incidence and mortality of HCC in China rank fourth and third among all cancers, respectively [2]. Therefore, HCC has already become a heavy worldwide health burden. Many studies have confirmed that the inactivation of multiple tumor suppressor genes, the activation of carcinogenic factors (including PI3K, K-Ras), the abnormal activation of multiple signaling pathways (including MAPK, JAK/STAT, NF-κB, and Wnt/β-catenin), the abnormal regulation of epigenetic genes (such as microRNAs), and autophagy are all involved in the occurrence and progression of HCC [3,4,5]. The major risk factors of HCC include chronic liver disease, cirrhosis, hepatitis virus infection (hepatitis B/C), obesity, drinking, diabetes, smoking, aflatoxin infection, and hereditary hemochromatosis [6]. Although modern surgery, ablation, chemotherapy, and radiotherapy improve the total survival of HCC, the clinical efficacy of the above methods is still limited by chemotherapy resistance. It is urgent to explore new drugs for the treatment of HCC. 

Pyroptosis is a kind of inflammatory cell death caused by microbial infection, which is activated by inflammasomes [7]. Endogenous and exogenous stimuli act on inflammatory corpuscles through different signal transduction pathways, activating caspase-1 and/or caspase-11, cleaving GSDMD, and then leading to the cleavage of interleukin IL-1β and IL-18 precursors, inducing the synthesis and release of a large number of inflammatory factors and adhesion molecules to induce pyroptosis [8]. Zhou et al. have found that iron-activated ROS could cause the oxidation and oligomerization of the mitochondrial outer membrane protein Tom20, and oxidized Tom20 recruits Bax to activate caspase-3, eventually triggering pyroptotic death by inducing GSDME cleavage [9]. 

As an important form of autophagy, mitophagy plays important roles in clearing damaged mitochondria and maintaining the homeostasis of the mitochondrial population in cells [10]. On the one hand, mitophagy promotes cancer cell survival through aerobic glycolysis or the Warburg effect. On the other hand, mitophagy induces cancer cell death via oxidative stresses, such as ROS [11]. BNIP3 (Bcl-2/adenovirus E1B 19-kDa-interacting protein 3) can promote the removal of damaged mitochondria through mitophagy and regulates both lethal and protective mitophagy in tumorigenesis [12]. 

Traditional Chinese medicine (TCM) is widely used for malignant tumor therapy based on clinical experience [13]. The formula of TCM with multiple components can improve the curative effects of complex symptoms in HCC by regulating different targets [14]. Mallotucin D (MLD) is a clerodane diterpenoid compound, derived from the root of *Croton crassifolius,* which is known as “jiguxiang” in China and distributed mainly in south and southwest China, Vietnam, Laos, and Thailand [15]. Accumulated studies have illustrated that the roots of *C. crassifolius* could be used to treat stomach aches, sternalgia, joint pain, pharyngitis, jaundice, and rheumatoid arthritis in China and treat cancer in Thailand [16,17]. However, there are no reports about MLD for HCC treatment. In this study, we aimed to determine the antitumor effects and explore the mechanisms by which MLD acts on HCC cells with a particular focus on ROS-meditated pyroptosis and mitophagy. We found that MLD could cause mitochondria damage inducing ROS overproduction, which activated mitophagy and pyroptosis to inhibit HepG2 cell proliferation.

## 2. Results

### 2.1. MLD Suppressed the Proliferation of HepG2 Cells and Angiogenesis of HUVECs 

**Figure 1:**
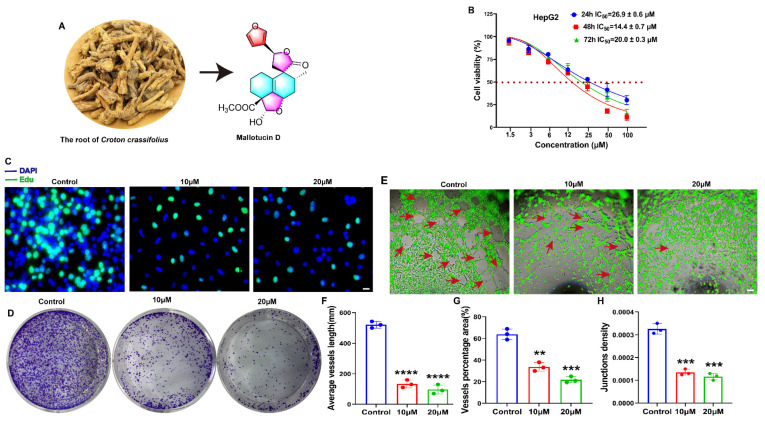
MLD suppressed the proliferation of HepG2 cells and angiogenesis of HUVECs. (**A**) Structure of MLD. (**B**) Cell viability of HepG2 cells after MLD treatment for 24 h, 48 h, and 72 h as determined by MTT assay. (**C**) The inhibitory effect on DNA synthesis in HepG2 cells was detected by Edu staining after treatment with MLD for 24 h. Scale bar: 100 μm. (**D**) The inhibitory effect of MLD on HepG2 cell colony formation. (**E**) The ability of HUVEC angiogenesis was inhibited in MLD-treated cells. Scale bar: 150 μm. Arrow: the angiogenesis-like structure. The statistics of tube-like structure length (**F**), vessel area (**G**), and junction density **(H)**. The results are representative of three independent experiments and are expressed as the mean ± SD. ** *p* < 0.01, *** *p* < 0.001, and **** *p* < 0.0001 compared with the control group.

The molecular structure of MLD is shown in Figure 1A. The detailed HR-ESI-MS, 1H NMR, 13C NMR, and HPLC were uploaded as Supplementary Materials. To study the effects of MLD on the vitality of HepG2 cells, MTT was performed. Our results showed that MLD significantly inhibited the cell viability of HepG2 cells in a dose-dependent manner. The IC_50_ values of MLD at 24 h, 48 h, and 72 h were 26.9 ± 0.6 μM, 14.4 ± 0.7 μM, and 20.0 ± 0.3 μM, respectively (Figure 1B). The results of the Edu assay indicated that the DNA synthesis of HepG2 cells was significantly inhibited in the MLD-treated group (Figure 1C). Moreover, MLD dramatically suppressed individual clones of HepG2 cells (Figure 1D). A tube formation assay was used to determine whether MLD has antiangiogenic effects on HUVECs. In the control group, HUVECs cultured showed a tube-like structure or tube network form, while MLD treatment impaired the tube formation of HUVECs (Figure 1E). In addition, MLD obviously inhibited the average tube-like structure length (Figure 1F), the tube-like structure percentage area (Figure 1G), and the tube-like structure junction density (Figure 1H) when compared with the control group. Thus, these results suggested that the proliferation of HepG2 cells was significantly inhibited by MLD, and MLD may possess the ability of antiangiogenesis in HCC.

### 2.2. MLD Decreased the Mitochondrial Membrane Potential, Inducing ROS Production in HepG2 Cells

**Figure 2:**
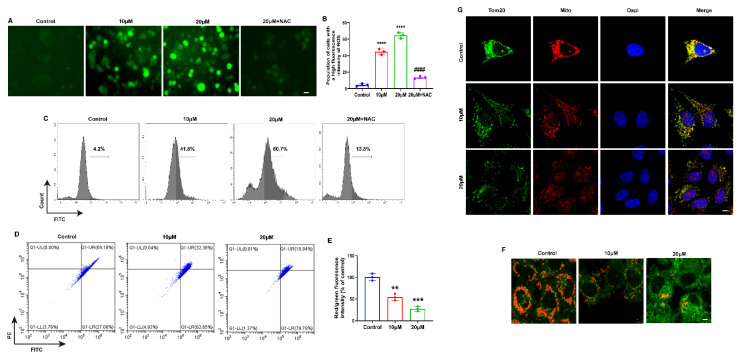
MLD Decreased the Mitochondrial Membrane Potential, Inducing ROS Production in HepG2 Cells. (**A**) DCFH-DA fluorescent probe detected showed that MLD significantly promoted ROS production after 24 h treatment in HepG2 cells. Scale bar: 75 μm. (**B**) Quantitative analysis of ROS production. (**C**) ROS production measured by flow cytometry. (**D**) The JC-1 fluorescence intensity was measured by flow cytometry. (**E**) Statistical results of the ratio of red and green fluorescence in HepG2 cells treated with MLD for 24 h. (**F**) Fluorescence photographs of mitochondrial membrane potential in HepG2 cells. Scale bar: 25 μm. (**G**) Fluorescence photographs of mitochondrial membrane protein TOM20 in HepG2 cells. Scale bar: 25 μm. The results are representative of three independent experiments and are expressed as the mean ± SD. ** *p* < 0.01, *** *p* < 0.001, and **** *p* < 0.0001 compared with the control group. ^####^ *p* < 0.0001 compared with MLD (20 μM) group.

Several studies have demonstrated that excess ROS can induce cancer cell death and inhibit cell proliferation [18]; we speculated that MLD may promote ROS production and thus induce HepG2 cell death. As presented in Figure 2A–C, the levels of cellular ROS were significantly upregulated in a dose-dependent manner by MLD treatment and downregulated by NAC, an ROS-scavenging reagent. The mitochondrial pathway is an important way to produce ROS. Therefore, we tested whether MLD could cause mitochondrial damage in HepG2 cells. The flow cytometry results showed that the mitochondrial membrane potential was significantly decreased after treatment with MLD in HepG2 cells (Figure 2D–F). As shown in Figure 2F, the red fluorescence, which indicates the J-aggregates in the mitochondrial matrix and is used to validate high mitochondrial membrane potential, was significantly decreased by MLD. In contrast, the green fluorescence, which indicates the J-aggregate monomers, was obviously increased by MLD. TOM20 targeted to the mitochondrial outer membrane and thereby involves the translocation of major protein precursors. TOM20 is an indicator of mitochondrial outer membrane integrity, and the decreased expression of TOM20 indicates mitophagy [19]. Moreover, the expression of TOM20 protein was significantly decreased in the MLD-treated group (Figure 2G). These results demonstrated that MLD could cause mitochondrial damage and decrease mitochondrial membrane potential to induce ROS production in HepG2 cells.

### 2.3. MLD Activated the Mitochondria Apoptosis of HepG2 Cells through Upregulating BNIP3 

**Figure 3:**
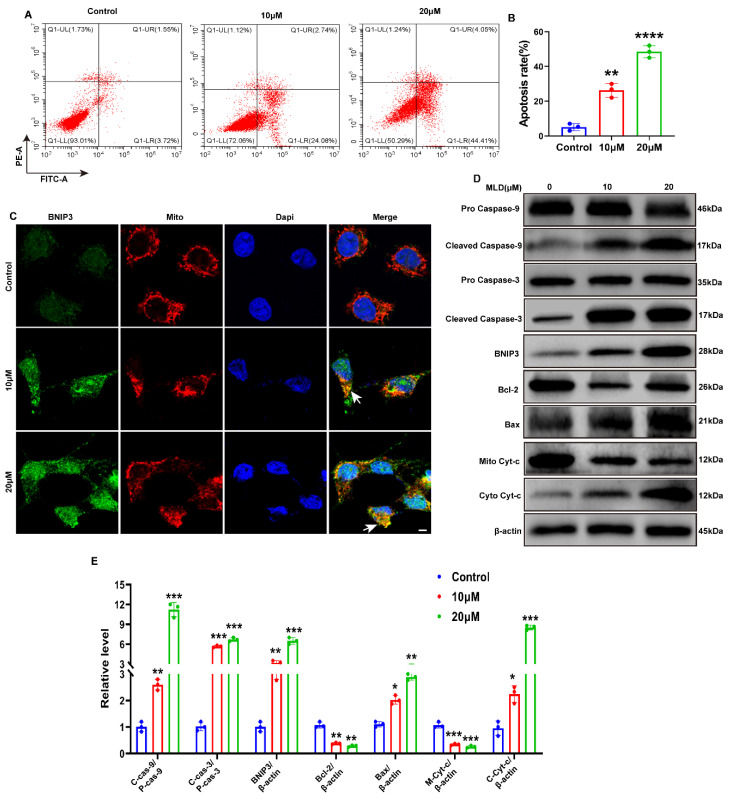
MLD induced the apoptosis of HepG2 cells by promoting the expression of BNIP3. (**A**) MLD induced apoptosis, as detected by Annexin V-FITC/PI double staining assay. (**B**) Quantitative analysis of the apoptosis. (**C**) The protein level of BNIP3 was detected by immunofluorescence. Scale bars: 50 μm. Arrow: the colocalization of BNIP3 and mitochondria. (**D**) Levels of the pro-caspase-9, cleaved caspase-9, pro-caspase-3, cleaved caspase-3, BNIP3, Bcl-2, Bax, Mito Cyt-c, and Cyto Cyt-c proteins in the different groups were analyzed by Western blotting. (**E**) The quantitative analysis of relative protein levels. The results are representative of three independent experiments and are expressed as the mean ± SD. * *p* < 0.05, ** *p* < 0.01, *** *p* < 0.001, and **** *p* < 0.0001 compared with the control group.

To explore whether MLD could induce HepG2 cell apoptosis, flow cytometry analysis was performed. We found that HepG2 cells underwent significant apoptosis after treatment with MLD (Figure 3A,B). Moreover, the expression of mitochondria apoptosis-related protein BNIP3 was dramatically increased in the MLD-treated group (Figure 3C,D). The Western blotting showed a dose-dependent increase in cleaved caspase-9, cleaved caspase-3 and Bax levels and a dose-dependent reduction in Bcl-2 levels after MLD treatment (Figure 3D). MLD also promoted the release of the cytochrome protein Cyt-c from mitochondria into the cytoplasm, resulting in the induction of HepG2 cell apoptosis through cleaving caspase-9 and caspase-3 (Figure 3D,E). Moreover, the cell viability of HepG2 was significantly increased after BNIP3 knockdown when compared with the group treated with MLD alone (Supplementary Figure S5). Taken together, these findings indicated that MLD promoted caspase-9/caspase-3-related mitochondria apoptosis in HCC cells by increasing the expression of BNIP3.

### 2.4. MLD Promoted HepG2 Cell Death via Activating GSDMD-Mediated Pyroptosis 

**Figure 4:**
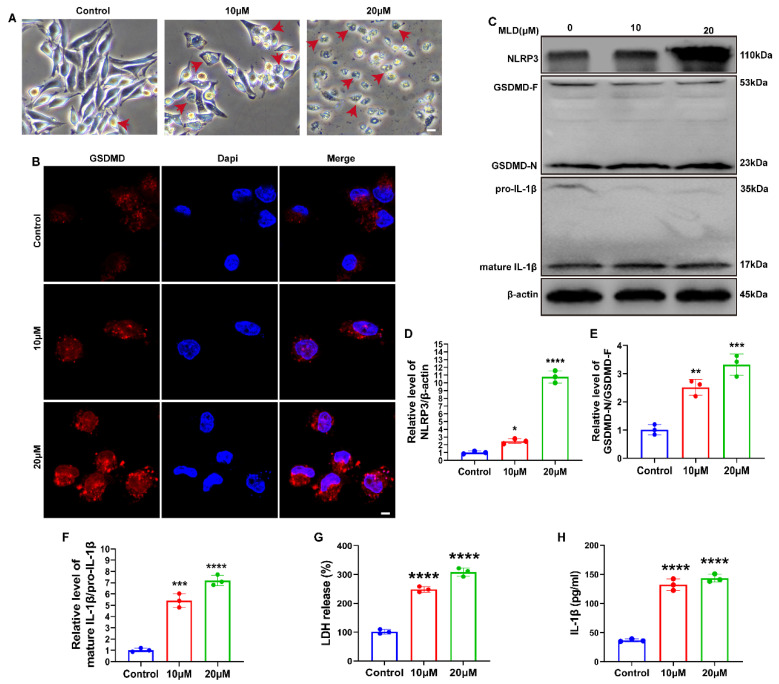
MLD promoted HepG2 cell death via activating GSDMD-mediated pyroptosis. (**A**) The morphological changes of HepG2 cells treated with MLD. Scale bar: 100 μm. Arrow: the pyroptosis cells. (**B**) The protein level of GSDMD was detected by immunofluorescence. Scale bars: 50 μm. (**C**) The protein levels of the NLRP3, GSDMD, and IL-1β in the different groups were analyzed by Western blotting. The quantitative analysis of NLRP3/β-actin (**D**), GSDMD-N/GSDMD-F (**E**), and mature IL-1β/pro-IL-1β (**F**). The levels of the LDH (**G**) and IL-1β (**H**) in the different groups were analyzed by ELISA. The results are representative of three independent experiments and are expressed as the mean ± SD. * *p* < 0.05, ** *p* < 0.01, *** *p* < 0.001, and **** *p* < 0.0001 compared with the control group.

As shown in Figure 4A, MLD-treated cells showed a pyroptotic cell phenotype, which presented large balloon-like bubbles on the plasma membrane. The fluorescence intensity of GSDMD was also significantly increased by MLD (Figure 4B). The Western blot data revealed that the protein levels of NLRP3, GSDMD-N/GADMD-F, and mature IL-1β/pro-IL-1β were all significantly upregulated in MLD-treated cells (Figure 4C–F). The release of proinflammatory factors, including LDH and IL-1β, was significantly elevated after MLD treatment, which indicated plasma membrane rupture and leakage (Figure 4G,H). In addition, the cell viability of HepG2 was significantly increased after GSDMD knockdown when compared with the group with MLD alone (Supplementary Figure S5). These data suggested that MLD promoted HepG2 cell pyroptosis by activating the GSDMD-mediated signaling pathway.

### 2.5. MLD Induced HepG2 Cell Mitophagy by Inhibiting the PI3K/AKT/mTOR Pathway

**Figure 5:**
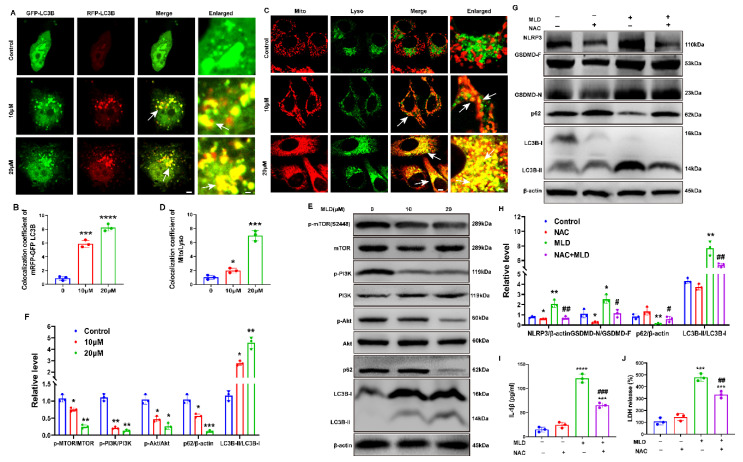
MLD induced HepG2 cell mitophagy by inhibiting PI3K/AKT/mTOR pathway. (**A**) Confocal detection of HepG2 cells transfected with mRFP-GFP-LC3B plasmid. Scale bar: 25 μm and 5 μm in merge and enlarged, respectively. Arrow: the colocalization of GFP-LC3B and RFP-LC3B. (**B**) Colocalization area statistics in A. (**C**) Mito staining and Lyso staining were used to analyze the fusion of mitochondria and lysosomes in HepG2 cells treated with MLD for 24 h. Scale bar: 25 μm and 5 μm in merge and enlarged, respectively. Arrow: the colocalization of mitochondria and lysosome. (**D**) Colocalization area statistics in C. (**E**) Levels of the PI3K, p-PI3K, AKT, p-AKT, mTOR, p-mTOR, P62, and LC3B proteins in the different groups were analyzed by Western blotting. (**F**) The quantitative analysis of relative protein levels of E. (**G**) Levels of the NLRP3, GSDMD, p62, and LC3B after treatment with MLD (20 μM) in the presence or absence of NAC (5 mM) for 24 h were measured by Western blotting. (**H**) The quantitative analysis of relative protein levels of G. The levels of IL-1β (**I**) and LDH (**J**) after treatment with MLD (20 μM) in the presence or absence of NAC (5 mM) for 24 h were measured by ELISA. The results are representative of three independent experiments and are expressed as the mean ± SD. * *p* < 0.05, ** *p* < 0.01, *** *p* < 0.001, and **** *p* < 0.0001 compared with the control group. ^#^ *p* < 0.05, ^##^ *p* < 0.01, and ^###^ *p* < 0.001 compared with MLD-treated group.

To evaluate the effects of MLD on autophagy, we transfected HepG2 cells with GFP-mRFP-LC3B plasmids and analyzed the immunofluorescence changes after MLD treatment. After transfection with GFP-mRFP-LC3B plasmids, the LC3B protein was labeled with mRFP red fluorescence and GFP green fluorescence. The GFP green fluorescence is unstable in the acid environment when the autophagosome fuses with the lysosome. Therefore, the cells exhibit red fluorescence when the autophagosome fuses with the lysosome. The red puncta indicate the autophagolysosome and the yellow puncta indicate autophagy. When autophagy is activated, both the red and yellow puncta will increase. In contrast, when autophagy is inhibited, the red and yellow puncta will decrease, or the red puncta will increase and the yellow puncta will decrease. As shown in Figure 5A, the red and yellow puncta markedly accumulated in HepG2 cells by MLD. Quantitatively, the number of autophagosomes per cell was increased following MLD treatment (Figure 5B). Next, we combined the LysoTracker Green and MitoTracker Red to explore whether MLD-induced cell autophagy was mitophagy, and we found that MLD significantly increased the colocalization of lysosomes and mitochondria (Figure 5C,D). As shown in Figure 5E,F, the ratio of LC3B-II/LC3B-I was significantly increased, while the expression of p62 was significantly decreased in the MLD-treated group, suggesting that autophagosomes were generated and accumulated in the cytoplasm after MLD treatment. Moreover, the ratios of p-PI3K/PI3K, p-AKT/AKT, and p-mTOR/mTOR in MLD treatment cells were all remarkably decreased compared to those in the control group. These results indicated that MLD induced HepG2 cell mitophagy by inhibiting the PI3K/AKT/mTOR pathway.

In order to study whether the pyroptosis and autophagy were activated via inducing ROS by MLD, the ROS-scavenging reagent NAC (5 mM) was used to stimulate MLD-treated HepG2 cells. As shown in Figure 5G,H, the Western blot results showed that the levels of NLRP3, GSDMD-N, and LC3B-II/LC3B-I were all increased and the level of p62 was decreased by MLD (20 μM). In contrast, the effects of MLD were significantly reversed by NAC. Interestingly, we also found the levels of IL-1β and LDH were all obviously decreased by NAC + MLD treatment when compared with the MLD-alone group (Figure 5I,J). As shown in Supplementary Figure S5C, the cell viability of HepG2 cells was significantly increased in the MLD plus NAC group when compared with MLD-alone group. These results suggested that MLD could activate HepG2 cell pyroptosis- and mitophagy-related signing pathways via upregulating ROS levels.

### 2.6. MLD Inhibited the HepG2 Cell Xenograft Tumor Growth In Vivo

**Figure 6:**
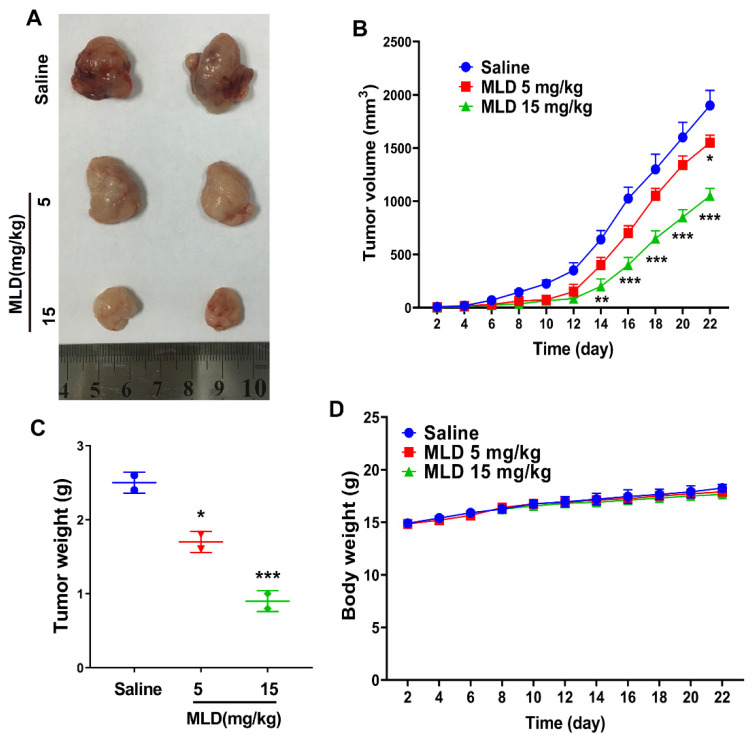
MLD inhibited the HepG2 cell xenograft tumor growth in vivo. (**A**) The photography of tumor tissues. (**B**) The tumor growth curve. (**C**) The statistics of tumor weight. (**D**) The body weight change curve of mice. The results are representative of three independent experiments and are expressed as the mean ± SD. * *p* < 0.05, ** *p* < 0.01, and *** *p* < 0.001 compared with the saline group.

**Figure 7:**
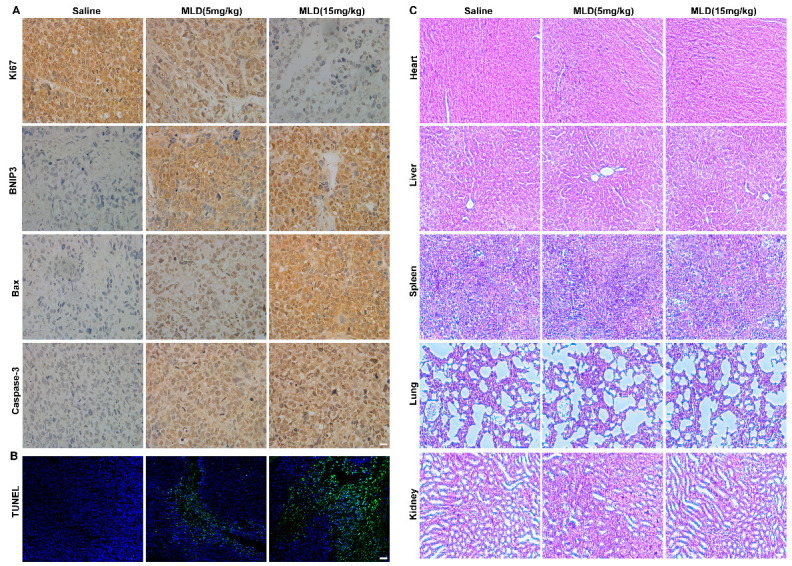
MLD promoted HepG2 cell death in vivo. (**A**) The levels of Ki67, BNIP3, Bax, and caspase-3 were detected by IHC. Scale bar: 100 µm. (**B**) The apoptosis of HepG2 cells as determined by TUNEL assay. Scale bar: 150 µm. (**C**) HE staining images of the main organs of mice (heart, liver, spleen, lung, and kidney). Scale bar: 100 µm. The results are representative of three independent experiments and are expressed as the mean ± SD.

We then developed a xenograft model in nude mice to investigate whether MLD inhibited HepG2 cell growth in vivo. As expected, MLD significantly suppressed tumor volume (Figure 6B) and tumor weight (Figure 6C) when compared with the control, as indicated by the images of the xenograft tumor (Figure 6A). There were no obvious changes in body weight after MLD treatment (Figure 6D). Furthermore, the IHC results showed that the level of ki67 was significantly downregulated, while the levels of apoptosis-related proteins, including BNIP3, Bax, and caspase-3, were all increased by MLD (Figure 7A). Moreover, the TUNEL assay also indicated that MLD promoted HepG2 cell death in vivo (Figure 7B). The toxicity of MLD to major organs was investigated by HE staining. As shown in Figure 7C, HE staining revealed that the histological morphology of different organs showed no obvious change after treatment with MLD. These results demonstrated that MLD may offer a safe and efficient therapy for HCC.

## 3. Discussion 

**Figure 8:**
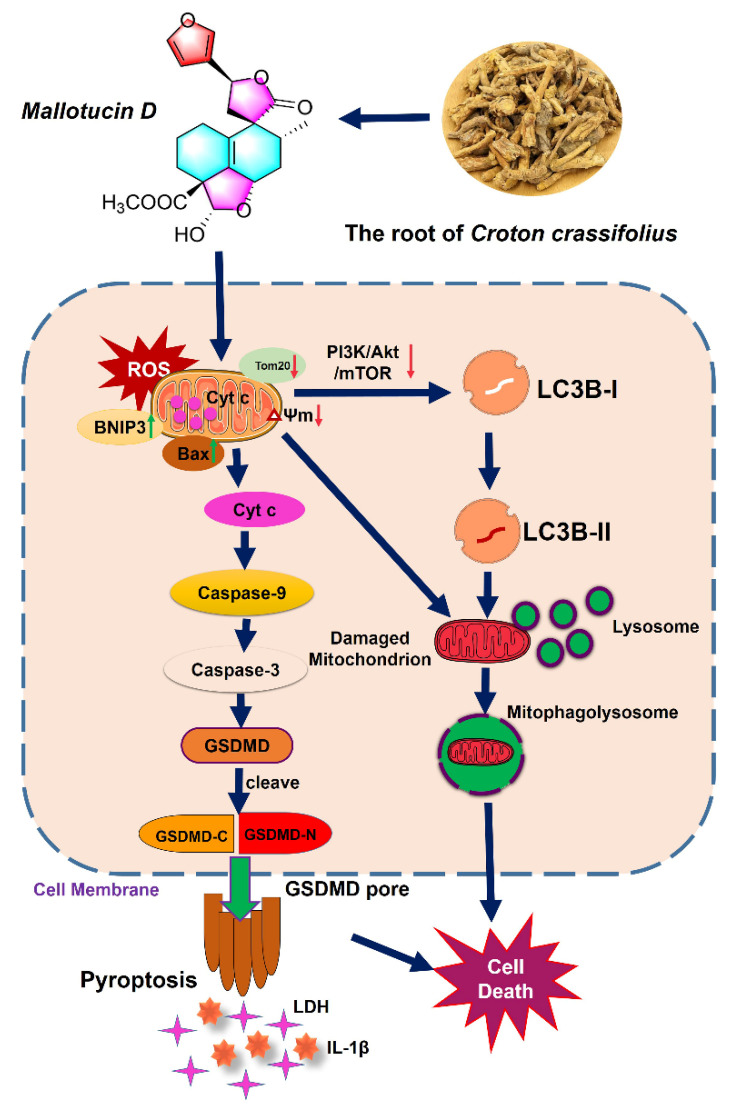
Model of the antitumor mechanism of MLD in HepG2 cells. MLD firstly caused mitochondrial damage and decreased the mitochondrial membrane potential, resulting in the production of ROS. The overproduction of ROS then increased BNIP3, inducing PI3K/AKT/MTOR-related mitophagy and GSDMD-related pyroptosis, leading to the induction of HepG2 cell death in vitro and in vivo.

Despite improved surveillance programs achieved globally, the 5-year survival of HCC patients is only 18%, and the prognosis of late-stage HCC remains dismal because of its high recurrence rates [20]. Previous phytochemical studies have demonstrated that the main active ingredients in *Croton crassifolius* are the derivatives of diterpenoids [21], which exhibit anti-inflammatory [22] and antiangiogenic [23] activities. In the present study, we found MLD, a clerodane-type diterpenoid from *Croton crassifolius*, significantly decreased the mitochondrial membrane potential and caused mitochondrial damage resulting in the production of ROS. The overproduction of ROS then increased BNIP3, inducing mitophagy and pyroptosis (Figure 8). Therefore, MLD could be developed as a useful bio-tool for elucidating the ROS-related autophagy and pyroptosis events in response to mitochondrial damage. Moreover, the IC50 values of MLD at 24 h, 48 h, and 72 h were 26.9 ± 0.6 μM, 14.4 ± 0.7 μM, and 20.0 ± 0.3 μM, respectively, which indicated that MLD possessed better antitumor activity when compared with most other small molecule compounds [24,25,26]. Therefore, MLD could be a good candidate drug for HCC therapy.

ROS include superoxide anion (O_2_^−^), hydrogen peroxide (H_2_O_2_), and hydroxyl radical (-OH). Most ROS are generated in mitochondria and are also closely related to mitochondria function [27,28]. Mitochondria is the site of oxidative phosphorylation, the energy supply of the cell, which generates ATP through a series of redox reactions. These redox reactions pump protons from the matrix space into the inner mitochondrial membrane and transfer electrons from the respiratory matrix to oxygen. On the one hand, ROS produced by mitochondria can increase cytotoxicity, promoting cell death. On the other hand, mitochondria themselves, as the main source of ROS production, are particularly vulnerable to ROS damage. These damages may cause adverse changes in the mitochondria, such as mitochondrial membrane permeability transition pore opening, mitochondrial permeability changes, and the oxidation of ATP to resolve phosphate coupling. These biological processes can promote mitochondria swelling, inducing apoptosis. Mitochondrial damage further intensifies intracellular ROS production, which can in turn exacerbate ER stress and mitochondrial damage, and forms a cycle [29]. Therefore, in our study, MLD directly promoted the mitochondria to intensify intracellular ROS production, and the overproduction of ROS in turn exacerbated the mitochondrial damage, which forms a cycle. 

ROS have been demonstrated to regulate the process involved in the initiation of apoptotic signaling [30]. Accumulating evidence indicates that many natural products can induce tumor cell apoptosis by increasing the level of ROS above a toxic threshold [18]. Mitochondria are known to produce significant amounts of endogenous ROS that contribute to intracellular oxidative stress [31]. Cytochrome c is important for ROS production by the generation of superoxide [32]. In our study, MLD was found to cause mitochondria damage, decrease the mitochondrial membrane potential, and promote cytochrome c release to the cytoplasm, which could induce ROS production. The increase in ROS caused by MLD-induced mitophagy and pyroptosis, inhibiting HepG2 cell proliferation. 

BNIP3 is usually located in the mitochondrial membrane, and overexpression of BNIP3 can disrupt the Bcl-2/Bax complex, leading to Bax liberation and Bax/Bak activation, which forms pores in the mitochondrial outer membrane and opens mPTP to promote the release of mitochondrial proteins, such as cytochrome c, to the cytosol, which initiates the activation of the caspase cascade and promotes the process of apoptosis [33]. In this study, we found that MLD could induce apoptosis by upregulating the expression level of proapoptotic factors, such as BNIP3, Bax, caspase-9 and caspase-3 cleavage, and downregulating the expression level of Bcl2. The xenograft tumor study also showed that MLD treatment significantly increased the protein levels of BNIP3, Bax, and caspase-3, and the TUNEL immunofluorescence was dramatically enhanced in the MLD-treated group. These results indicated that MLD could induce cell apoptosis and thus inhibit HCC growth both in vitro and in vivo. However, the biomolecules in mitochondria to which MLD directly binds and that perturb its function in exerting anticancer effects are still not sufficiently clear; surface plasmon resonance (SPR) could be used to find the directly binding biomolecules, and genomics or proteomics may be useful to explore the mechanism of action of MLD in future work. 

Pyroptosis is a new form of programmed cell death, and many anticancer drugs can induce pyroptosis to inhibit cancer growth [34]. Our data showed that MLD treatment induced the pyroptosis of HepG2 cells by activating the NLRP3 inflammasome and GSDMD cleavage, leading to the release of LDH and IL-1β. Xiaowei Zhang et al. have found that miltirone could increase the protein levels of BAX, cleaved caspase-9, and cleaved caspase-3 to induce Hepa1-6 cell pyroptosis, and the caspase-3 siRNA and inhibitor could inhibit miltirone-induced pyroptosis [35]. The group of Ling Liu has reported that JS-K can decrease mitochondrial membrane potential, promote cyt-c release from mitochondria, and activate cleaved caspase-9/3 [36]. In our study, we found that the expression level of BNIP3 in mitochondria was significantly increased, while the mitochondrial ΔΨm and Tom20 proteins were dramatically downregulated by MLD, leading to the release of cyt-c from mitochondria to the cytoplasm. The cytoplasm cyt-c then activated the downstream caspase-9 and cleaved caspase-3 to cleave GSDMD to induce pyroptosis. 

BNIP3 is well known as a mitophagy receptor that can activate mitophagy to clear damaged mitochondria to inhibit several types of tumor growth [11]. Our results showed that the number of autophagosomes and the colocalization of lysosomes and mitochondria were increased following MLD treatment. The PI3K/AKT/mTOR pathway plays a key role in autophagy [37]. The mTOR pathway occurred in up to 50–60% of HCC cases and is associated with recurrence and poor prognosis [38]. In lung cancer, gefitinib and tripchlorolide induce lung cancer cell autophagy and apoptosis via blockade of the PI3K/AKT/mTOR pathway [39]. Our results demonstrated that MLD induced mitophagy by inhibiting the PI3K/AKT/mTOR signaling pathway. Generally speaking, the overproduction of ROS may induce autophagy by increasing the accumulation of LC3B and the formation of autophagy via inhibiting the PI3K/AKT/MTOR signaling pathway [40]. NAC is a reducing agent and can react with and scavenge ROS, but it will not inhibit the source of ROS production [41]. In our study, the level of ROS was increased to 60.7% by 20 μM MLD, while it was obviously reversed to 13.8% by NAC. Therefore, we speculated that most of the ROS induced by MLD were scavenged by NAC; however, a small amount of newly produced ROS escaped NAC scavenging. In the present study, the ROS-scavenging reagent NAC increased the p62 level; reversed MLD-induced NLPR3, GSDMD, and LC3-II expression; and decreased the levels of IL-1β and LDH, suggesting that the activation of ROS was required for MLD-induced mitophagy and pyroptosis in HepG2 cells. 

## 4. Materials and Methods 

### 4.1. Extraction and Isolation

The dried and powdered roots of *Croton crassifolius* (9.0 kg) were extracted with 95% ethanol at room temperature, and the concentrated extract was partitioned between water and chloroform. The chloroform part (390 g) was fractionated using a silica gel column (petroleum ether/ethyl acetate, 100:1 → 100:50) to afford fractions A–G. FrE was further separated by a silica gel column (petroleum ether/ethyl acetate, 80:20 → 50:50) to give fractions E1–E6. Finally, MLD (85 mg) was obtained from Fr.E5 via a preparative HPLC (60% methanol). The purity of MLD was confirmed to be 98% by HPLC.

### 4.2. General Experimental Procedures 

Preparative HPLC was performed using a COSMOSIL C18 preparative column (5 μm, 20 × 250 mm). HRESIMS data were acquired using an Agilent 6210 ESI/TOF mass spectrometer (NYSE: A, MA, USA). Optical rotations were measured using a JASCO P-1020 digital polarimeter (JASCO, Tokyo, Japan). UV spectra were measured using a JASCO V-550 UV/vis spectrophotometer (JASCO, Tokyo, Japan). A JASCO FT/IR-480 plus FT-IR spectrometer was used for scanning the IR spectra with KBr pellets (JASCO, Tokyo, Japan). The 1D and 2D NMR spectra were obtained on a Bruker AV-600 spectrometer with TMS as internal standard. All chemical reagents were purchased from Tianjin Damao Chemical Company (Tianjin, China).

### 4.3. Cell Lines and Reagents 

The human HCC cell line HepG2 was obtained from the American Type Culture Collection (ATCC, Manassas, VA, USA). Cells were cultured in DMEM medium (GIBCO) under standard conditions with 10% fetal bovine serum (FBS) and 5% CO_2_ at 37 °C in a humidified incubator (ESCO). 

### 4.4. MTT Assay

Cells (8 × 10^3^/well) were seeded in 96-well plates in a medium containing 10% FBS. After 24 h, different concentrations of MLD were added to the medium. After 24 h, 48 h, and 72 h treatment, a final concentration of 0.5 mg/mL MTT (Sigma Chemical Co., St. Louis, MO, USA) was added to cells, and the plate was incubated for 4 h at 37 °C, followed by the addition of DMSO stop solution. Absorbance was measured at 490 nm using a spectrophotometer. Cell viability inhibition rate was calculated using the following formula: relative cell viability (%) = (OD_treated_ − OD_start_)/(OD_control_ − OD_start_) ×100%. 

### 4.5. ROS Assay

An ROS assay with 2′,7′-dichlorofluorescein diacetate (DCFH-DA) was used to test the cellular ROS levels. Cells were cultured in a 6-well plate and treated with MLD for 24 h. After washing with PBS, the cells were stained with DCFH-DA (10 µM) for 30 min at 37 °C in the dark. The ROS levels were determined using a fluorescence microscope and flow cytometer (BD, Franklin Lakes, NJ, USA).

### 4.6. ELISA Assay

The lactate dehydrogenase (LDH) and IL-1β levels were measured using a CytoTox96 LDH-release kit (Promega, Madison, WI, USA) and a QuantiCyto IL-1β ELISA kit (Neobioscience, Shenzhen, China), according to the manufacturer’s instructions. The absorbance value at 450 nm was then measured. 

### 4.7. Immunofluorescence of Tom20, BNIP3, and GSDMD

Cells (1 × 10^3^ cells/well) were seeded into a 24-well plate with a chamber slide. MLD was added to the cells and incubated for 24 h. Cells were fixed using 4% formaldehyde for 15 min, permeabilized with 0.5% triton for 10 min, blocked with 5% BSA for 1 h, and incubated with primary antibodies overnight at 4 °C, and specific proteins were detected using secondary antibodies. After DAPI staining was performed, images were observed using immunofluorescence microscopy (Nikon, Tokyo, Japan). The signal intensities in the samples under different conditions were analyzed by using ImageJ.

### 4.8. Western Blot Assay 

Cells were lysed using the PRO-PREPTM Protein Extraction solution (iNtRon Biotech, Seongnam-Si, Republic of Korea). Total proteins were transferred to a polyvinylidene difluoride membrane and blocked with 5% skimmed milk for 1 h. Specific proteins were detected with primary antibodies: NLRP3 (A12694), GSDMD (A20197), IL-1β (A1112), caspase-3 (A19654), caspase-9 (A11451), BNIP3 (A5683), Cyt-c (A1561), Bax (A19684), Bcl-2 (A19693), β-actin (AC026), p-mTOR (S2448) (AP0115), mTOR (A2445), p-PI3K (AP0845), PI3K (A4992), p-Akt (AP0637), Akt (A17909), P62 (A19700), and LC3B (A19665), which were all bought from ABclonal Technology Co. Ltd., Wuhan, China. The blots were then treated with horseradish peroxidase-conjugated secondary antibodies. Bands were visualized using the ECL reagent and FluorChem M system (ProteinSimple, SFO, San Jose, CA, USA)

### 4.9. Colony Formation

Cells (5 × 10^2^ cells/well) were seeded into a 6-well culture plate and then exposed to different concentrations of MLD for 9 days, and the fresh MLD was changed every three days. The colonies were fixed with 4% paraformaldehyde for 15 min and incubated with crystal violet solution (0.1%) for 30 min. The stained colonies were counted and photographed by using a Nikon microscope.

### 4.10. Edu Staining Assay

Cells (1 × 10^3^ cells/well) were seeded into a 24-well plate with a chamber slide. MLD was added to the cells and incubated for 24 h. Cells were fixed using 4% formaldehyde, washed with PBS, and permeabilized with 0.5% triton for 10 min. After washing with PBS, the cells were stained with Edu and DAPI, and the images were observed using immunofluorescence microscopy (Nikon).

### 4.11. Apoptosis Assay

Cells (5× 10^5^ cells/well) were seeded in a 6-well plate and treated with MLD. Annexin V and PI (4A BIOTECH, Beijing, China) were added to the cells and incubated for 15 min in the dark. Apoptotic cells were quantified using flow cytometry (Beckman FACSCalibur Flow Cytometer, Brea, CA, USA).

### 4.12. Tube Formation Assay

The matrigel was thawed at 4 °C, and the 96-well plate was coated with 100 μL of matrigel per well and kept at room temperature for 30 min to allow the gelling of the matrigel. Then 100 μL of the cell mixture, containing 1000 cells and MLD, was plated into each matrigel-coated well. After 4 h, the cells were stained for 2 min with Calcein AM (40719ES50, Yeasen, Shanghai, China). Images were captured using a Nikon microscope. The tube formation ability was analyzed using Image J software.

### 4.13. JC-1 Staining Assay

The enhanced mitochondrial membrane potential assay kit with JC-1 was purchased from Beyotime Biotechnology, Shanghai, China. The experimental steps followed the instruction. Firstly, the JC-1 dyeing solution was prepared; then, the cells were incubated with JC-1 dyeing solution for 30 min at 37 °C to stain the mitochondrial membrane. The cells were washed with PBS and subjected to microscopy and flow cytometry. The JC-1 fluorescent probe is a fluorescent dye that forms aggregates in the mitochondria with a red emission (~590 nm) that revert to monomers with a green emission (~526 nm) when they start to depolarize. We chose the PE channel for red emission and the FITC channel for green emission when using flow cytometry.

### 4.14. MitoTracker Red CMXRos Staining and LysoTracker Green Staining

The MitoTracker Red CMXRos Staining and LysoTracker Green Staining kits were purchased from Beyotime Biotechnology, Shanghai, China. According to the manufacturer’s recommendation, the cells were incubated with MitoTracker Red CMXRos dyeing solution for 30 min at 37 °C. The cells were washed with PBS and subjected to microscopy.

### 4.15. The HepG2 Cell Xenograft Tumor Study

The 5-week-old male BALB/c nude mice were obtained from Guangzhou animal experimental center (Guangdong, China) and raised in a specific-pathogen-free facility at 22 °C under 12 h light/dark cycles. The animal study was reviewed and approved by the Administrative Committee on Animal Research of the Shenzhen International Graduate School, Tsinghua University (No. 16, 2018). HepG2 cells (5 × 10^6^ cells in 100 μL PBS/mouse) were infused subcutaneously into the flanks of nude mice. The mice were treated with MLD once every two days by intraperitoneal injection. Body weight and tumor sizes were measured and recorded every two days. The primary tumor volume was calculated using the following formula: (length) × (width)^2^ × 0.5. After 15 days of treatment, the mice were sacrificed to collect tumors, blood, and other viscera for further research.

### 4.16. HE Staining and Immunohistochemistry (IHC)

The processing for histological analysis was performed following a standard protocol, as described previously [42]. Briefly, tumor tissues were fixed in 10% buffered formalin and paraffin-embedded. Tumor sections were stained with monoclonal antibodies. Subsequent operations were performed in accordance with standard immunohistochemical protocols utilizing predetermined optimal primary antibody concentrations, SAB detection, and 3′3′-di-aminobenzidine tetra-hydrochloride (DAB). HE staining was performed on paraffin-embedded tumor tissues with a slice thickness of 6 μm for histological study.

### 4.17. TUNEL Staining

TUNEL staining (Beyotime Biotechnology, Shanghai, China) was performed to detect cell apoptosis in the tumor tissue. After dewaxing and rehydrating, tumor tissue sections were washed with PBS buffer and permeabilized with Proteinase K (0.02 μg/μL). According to the manufacturer’s recommendation, these slices were incubated with a TUNEL staining solution. Images were captured using a Nikon microscope.

### 4.18. The Transfection of shRNA

The plasmids were transfected with Lipofectamine 3000 transfection reagent (LTI, Carlsbad, USA) according to the manufacturer’s protocol. Briefly, before transfection, the medium was replaced with Opti-MEM medium. The ratio of plasmid to Lipofectamine 3000 was selected as 1:2.5 (mass:volume). The BNIP3 or GSDMD shRNA plasmids and Lipofectamine 3000 were separately diluted with Opti-MEM medium and incubated at room temperature for 5 min. Lipofectamine 3000 was then slowly added to the diluted plasmids, incubated at room temperature for 10 min, and then added to the cells. After transfection for 12 h, the medium was removed, and the complete medium containing 10% FBS was added to the cells. The BNIP3 and GSDMD shRNA were all purchased from OriGene Technologies Inc. (Rockville, MD, USA).

### 4.19. Serum Biochemistry and Hematological Assessments

In brief, the collected blood was stored at 4 °C overnight and then centrifuged (3000 rpm, 15 min) to obtain the serum. One hundred microliters of serum was taken out and added to 200 μL of PBS for blood biochemistry examination and hematological assessments. 

### 4.20. Statistical Analysis

All data are expressed as mean ± SD. One-way ANOVA and Tukey multiple comparison tests (Graphpad Software, Inc., San Diego, CA, USA) were used for group comparison. * *p* < 0.05, ** *p* < 0.01, *** *p* < 0.001, and **** *p* < 0.0001 were considered statistically significant.

## 5. Conclusions

The present study has demonstrated that MLD induced mitophagy and pyroptosis and thus inhibited HepG2 cell growth in vitro and in vivo. MLD caused mitochondrial damage, resulting in the production of ROS; decreased the mitochondrial ΔΨm; and released cyt-c to the cytoplasm, activating caspase-9/cas.pase-3/GSDMD-mediated pyroptosis. In addition, MLD could activate mitophagy by blocking the PI3K/AKT/mTOR signaling pathway. MLD might be developed as a candidate agent for HCC therapy. 

## Data Availability

Not applicable.

## Supplementary Materials

The following supporting information can be downloaded at: https://www.mdpi.com/article/10.3390/ijms232214217/s1. The detailed HR-ESI-MS, 1H NMR, 13C NMR, and HPLC are uploaded as Supplementary Materials.
