# Correction: Dai et al. Mallotucin D, a Clerodane Diterpenoid from *Croton crassifolius*, Suppresses HepG2 Cell Growth via Inducing Autophagic Cell Death and Pyroptosis. *Int. J. Mol. Sci.* 2022, *23*, 14217

**DOI:** 10.3390/ijms27073327

**Published:** 2026-04-07

**Authors:** Xiaoyong Dai, Fen Sun, Kexin Deng, Gaoyang Lin, Wenjing Yin, Huaqing Chen, Dongye Yang, Kewei Liu, Yubo Zhang, Laiqiang Huang

**Affiliations:** 1 Precision Medicine and Healthcare Research Center, Center for Biotechnology and Biomedicine, Shenzhen Key Laboratory of Gene and Antibody Therapy, State Key Laboratory of Chemical Oncogenomics, State Key Laboratory of Health Sciences and Technology, Tsinghua-Berkeley Shenzhen Institute (TBSI), Institute of Biopharmaceutical and Health Engineering, Shenzhen International Graduate School, Tsinghua University, Shenzhen 518055, Chinafensun2017@126.com (F.S.);; 2 School of Life Sciences, Tsinghua University, Beijing 100084, China; 3 Department of Chemistry, Tsinghua University, Beijing 100084, China; 4 Guangdong Clinical Translational Center for Targeted Drug, Department of Pharmacology, School of Medicine, Jinan University, Guangzhou 510632, China; 5 Division of Gastroenterology and Hepatology, The University of Hongkong-Shenzhen Hospital, Shenzhen 518055, China

In the original publication [1], there was a mistake in Figure 7. In this figure, we inadvertently used the representative duplication image of Bax from the saline group for Caspase-3. The authors sincerely apologize for this unintentional error. The corrected Figure 7 and figure caption appears below. The authors state that the scientific conclusions are unaffected. This correction was approved by the Academic Editor. The original publication has also been updated.

**Figure 7:**
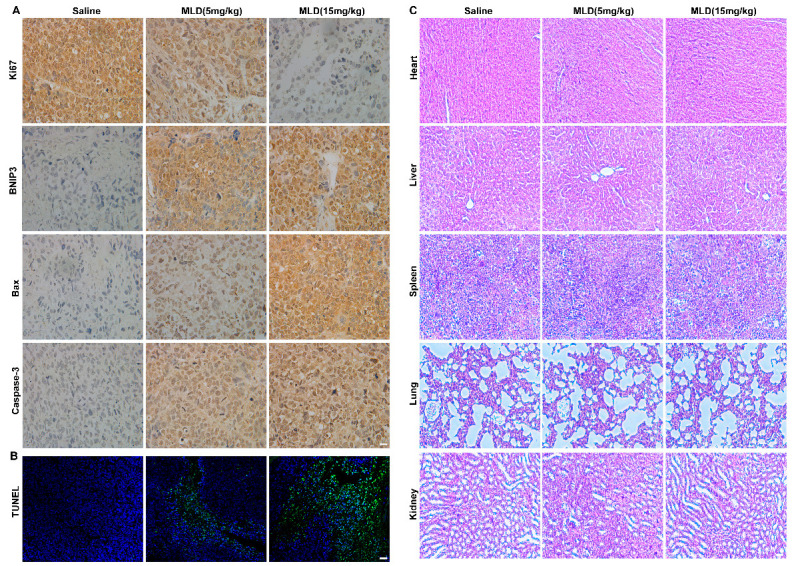
MLD promoted HepG2 cell death in vivo. (**A**) The levels of Ki67, BNIP3, Bax, and caspase-3 were detected by IHC. Scale bar: 100 µm. (**B**) The apoptosis of HepG2 cells as determined by TUNEL assay. Scale bar: 150 µm. (**C**) HE staining images of the main organs of mice (heart, liver, spleen, lung, and kidney). Scale bar: 100 µm. The results are representative of three independent experiments and are expressed as the mean ± SD.
