# Supplementary material for: Mallotucin D, a Clerodane Diterpenoid from Croton crassifolius, Suppresses HepG2 Cell Growth via Inducing Autophagic Cell Death and Pyroptosis
Source: Int J Mol Sci. 2022 Nov 17;23(22):14217. doi: 10.3390/ijms232214217 (PMC9698996; doi:10.3390/ijms232214217)
Supplement: Supplementary file 1 [file ijms-23-14217-s001.zip › Supplementary Materials-Round 6.pdf]

Supplementary Materials

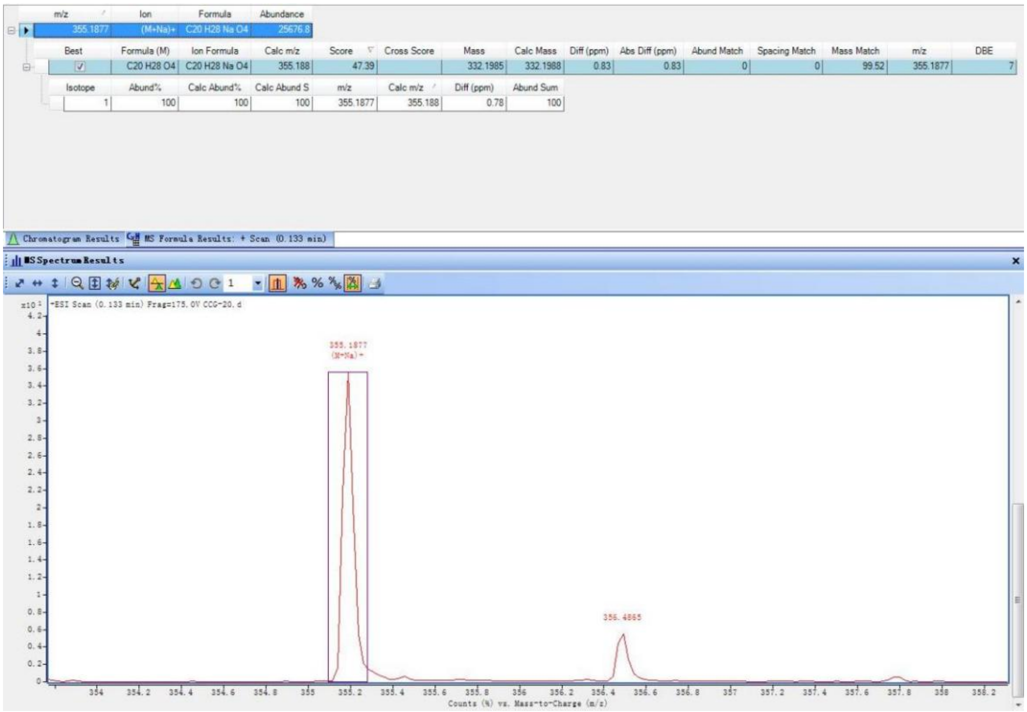

Supplementary Figure 1. HR-ESI-MS of MLD. HR-ESI-MS m/z: 413.1570 [M + Na]+ (calcd for C21H26O7Na, 413.1571).

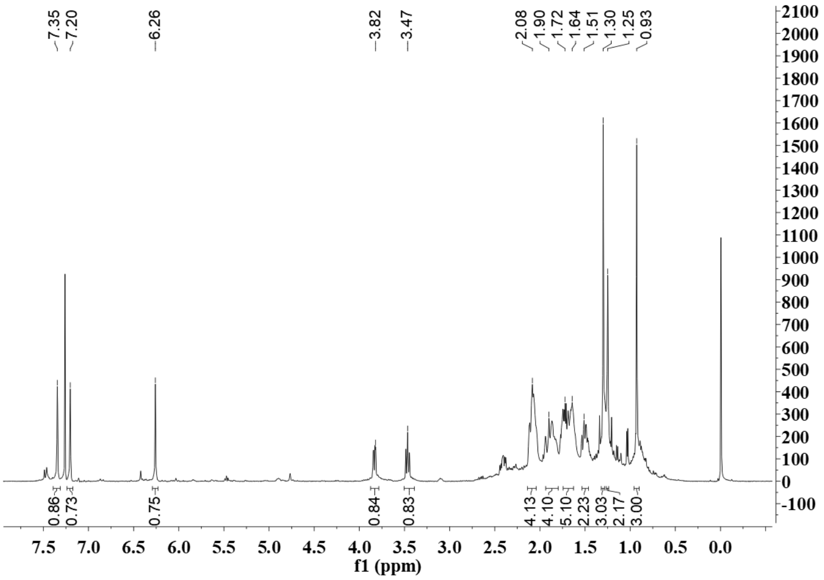

Supplementary Figure 2. ^{1}H NMR spectrum of MLD (400 MHz, $CDCl_3$).

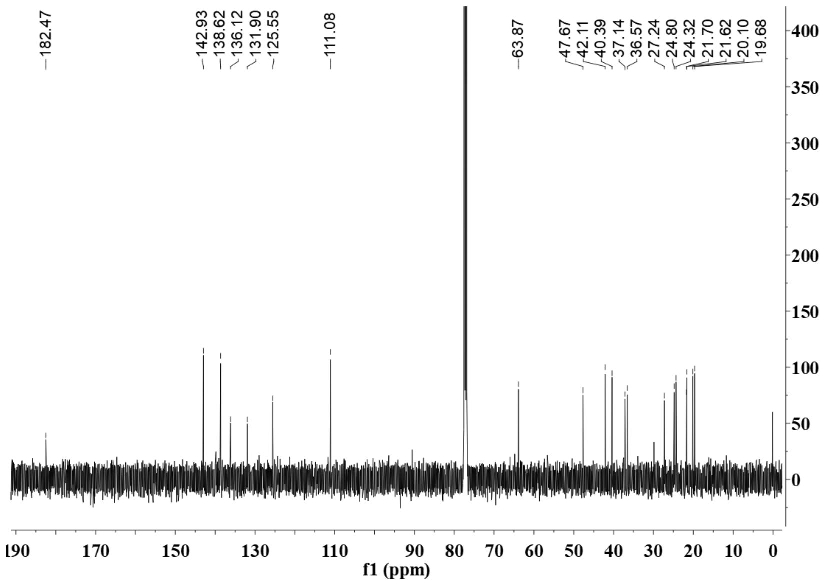

Supplementary Figure 3. ^{13}C **NMR spectrum of MLD (100 MHz,** $CDCl_3$**).**

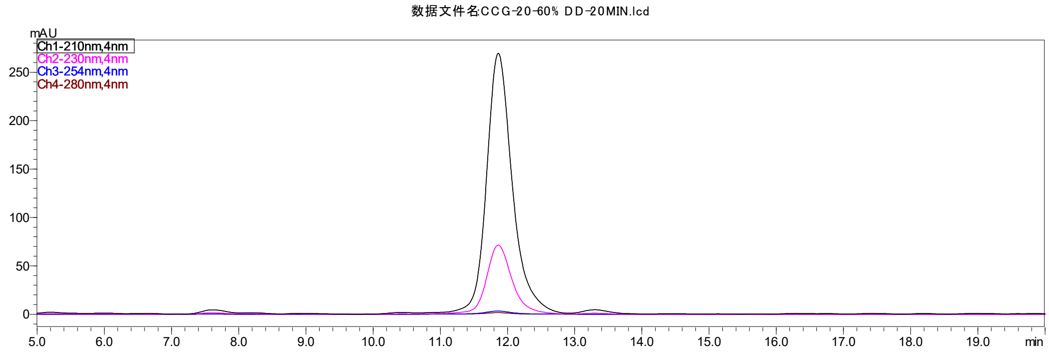

Supplementary Figure 4. HPLC of MLD (60%MeOH-H_2O**).**

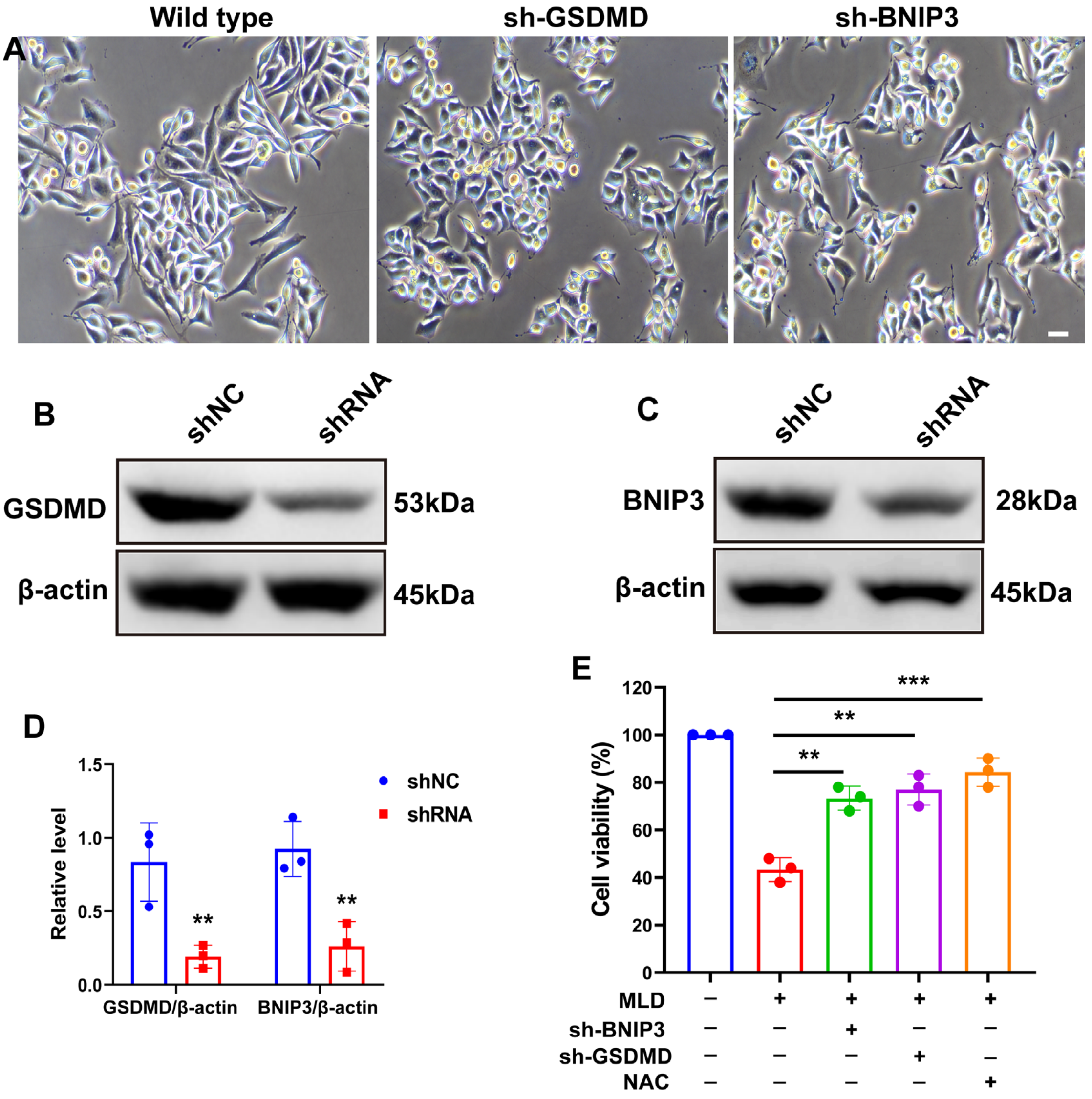

Supplementary Figure 5. Cell viability of HepG2 cells after different treatment by using MTT assay. **(A)** The morphological changes of HepG2 cells treated with shRNA. Scale bar: 100 μm. The levels of GSDMD **(B)** and BNIP3 **(C)** after shRNA transfection. **(D)** The quantitative analysis of relative protein levels. **(E)** The cell viability of HepG2 cells after different treatment by using MTT assay. The results are representative of three independent experiments and are expressed as the mean ±SD. **p<0.01 and ***p<0.001 compared with the control group.

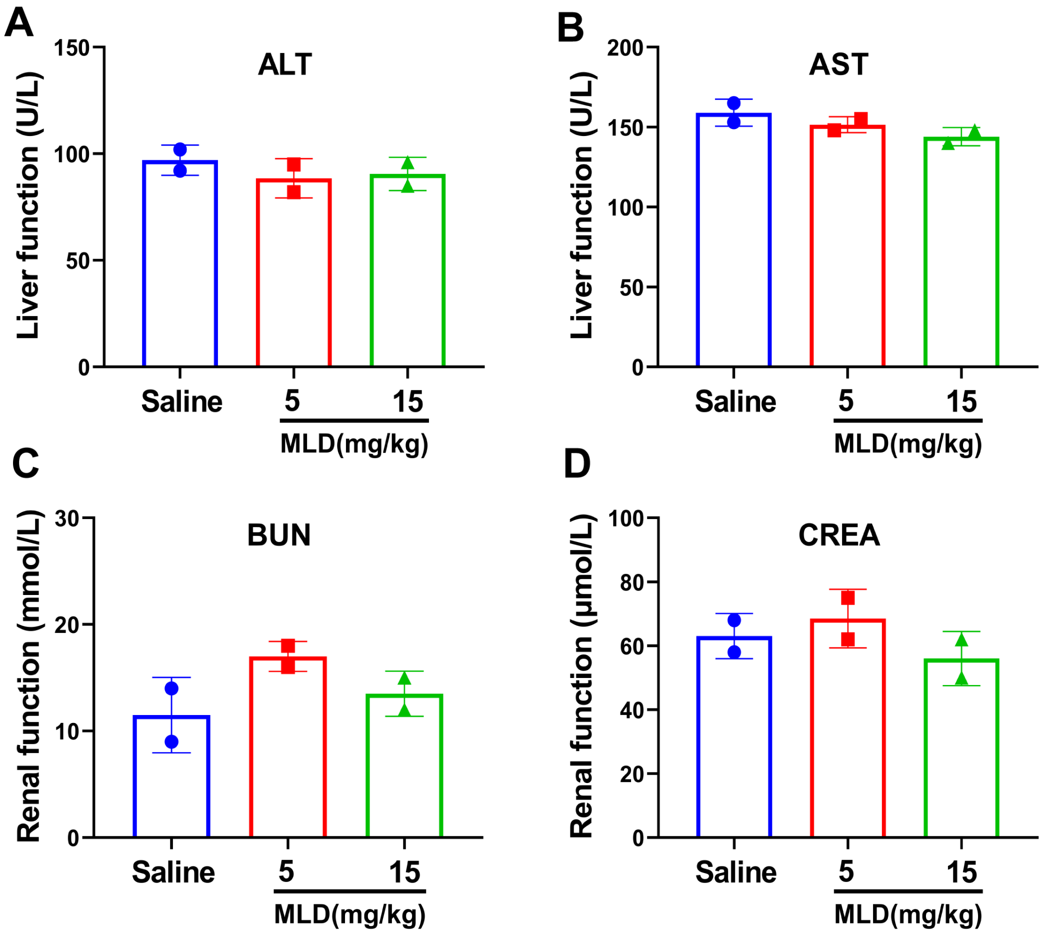

Supplementary Figure 6. Serum biochemistry assessment on the mice treated with MLD. The ALT **(A)** and AST **(B)** indicators of liver function. The BUN **(C)** and CREA **(D)** indicators of renal function. The results are representative of three independent experiments and are expressed as the mean ±SD. $^{**}p<0.01$ and $^{***}p<0.001$ compared with the control group.

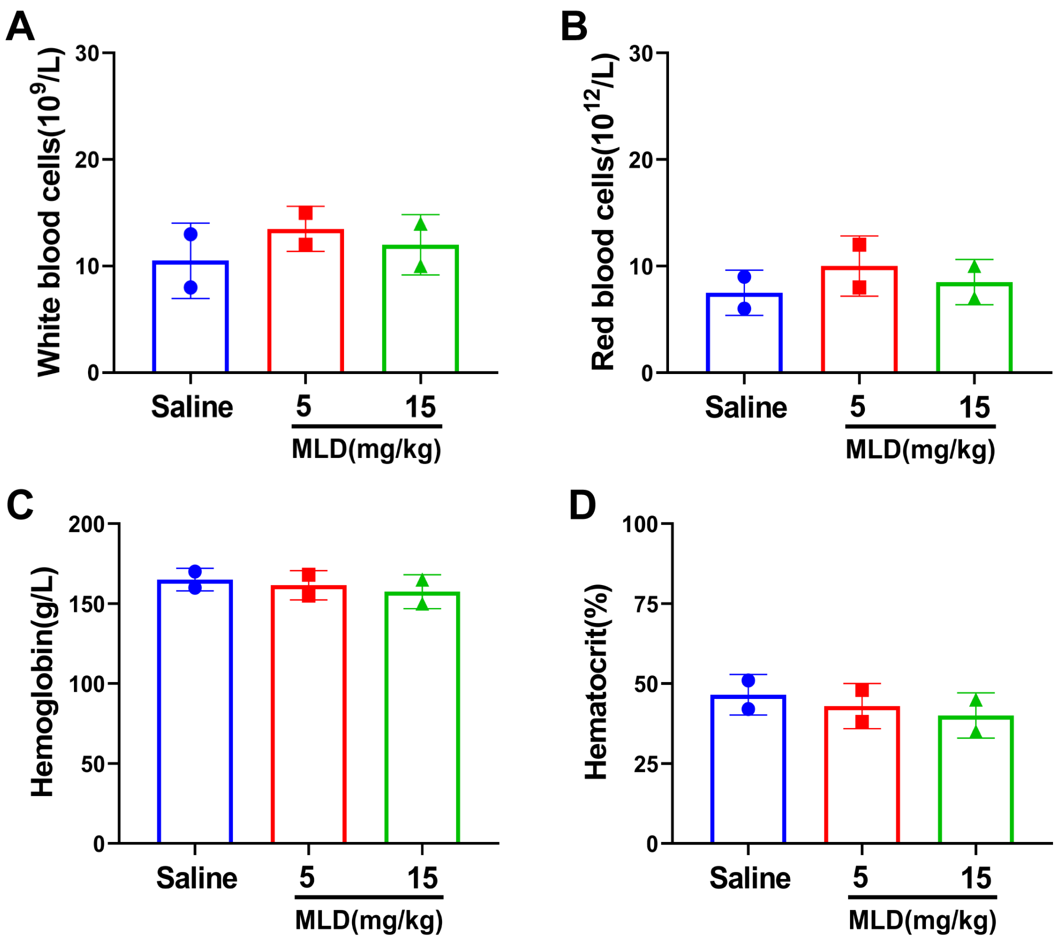

Supplementary Figure 7. Hematological assessment on the mice treated with MLD. The changes of white blood cells **(A),** red blood cells **(B),** hemoglobin (C) and hematocrit (D). The results are representative of three independent experiments and are expressed as the mean ±SD. ** $p<0.01$ and *** $p<0.001$ compared with the control group.

Supplementary Table 1 ^1H and ^{13}C NMR data of MLD ($CDCl_3$, δ in ppm, *J* in Hz)[a]

Position	**CCG-20**	
	δ_H	δ_C
1	α 2.25 (m), β 1.84 (m)	24.9
2	1.91 (m)	19.8
3	α 1.95 (m), β 1.55 (m)	25.8
4	-	58.5
5	-	134.7
6	4.52 (m)	75.6
7	α 2.24 (m), β 1.90 (m)	36.3
8	2.00 (m)	36.4
9	-	53.0
10	-	133.4
11	α 2.77 (dd, 13.9, 8.3), β 2.28 (m)	39.5
12	5.46 (m)	72.8
13	-	124.9
14	6.42 (s)	108.2
15	7.45 (s)	144.4
16	7.49 (s)	139.8
17	1.10 (d, 6.8)	16.6
18	-	173.6
19	5.48 (s)	100.3
20	-	177.6
18-OCH_3	3.73 (s)	52.7
