# Supplementary material for: Mallotucin D, a Clerodane Diterpenoid from Croton crassifolius, Suppresses HepG2 Cell Growth via Inducing Autophagic Cell Death and Pyroptosis
Source: Int J Mol Sci. 2022 Nov 17;23(22):14217. doi: 10.3390/ijms232214217 (PMC9698996; doi:10.3390/ijms232214217)
Supplement: Supplementary file 1 [file ijms-23-14217-s001.zip › Supplementary Materials-Western Blots.pptx]

## Slide 1
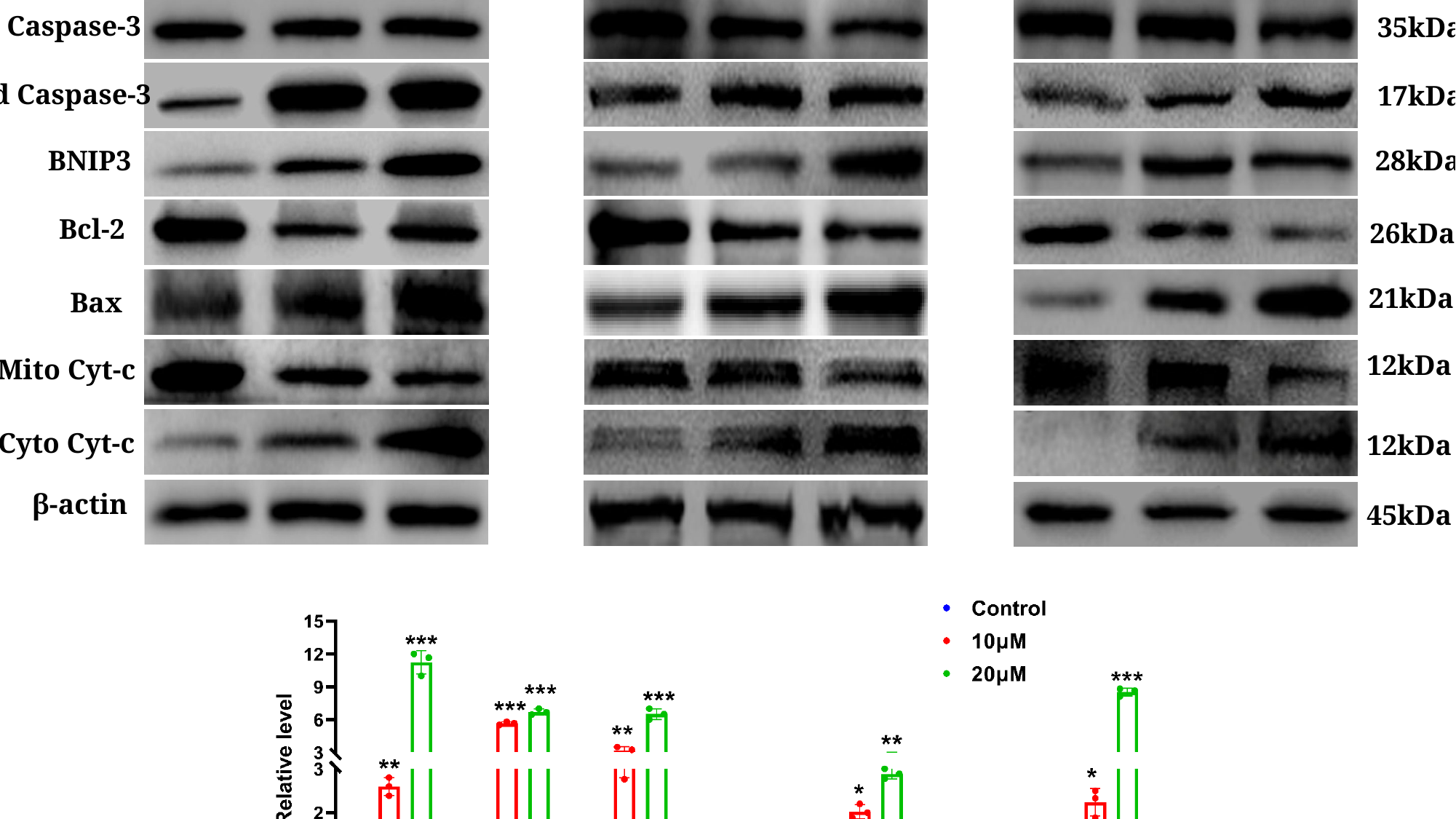

1#
2#
3#
10
10
10
0
0
0
20
20
20
MLD(μM)
46kDa
Pro Caspase-9
Cleaved Caspase-9
17kDa
Pro Caspase-3
35kDa
Cleaved Caspase-3
17kDa
BNIP3
28kDa
Bcl-2
26kDa
21kDa
Bax
12kDa
Mito Cyt-c
Cyto Cyt-c
12kDa
β-actin
45kDa
C-Cyt-c/
β-actin
Bcl-2/
β-actin
M-Cyt-c/
β-actin
BNIP3/
β-actin
Bax/
β-actin
C-cas-3/
P-cas-3
C-cas-9/
P-cas-9
Figure 3D

## Slide 2
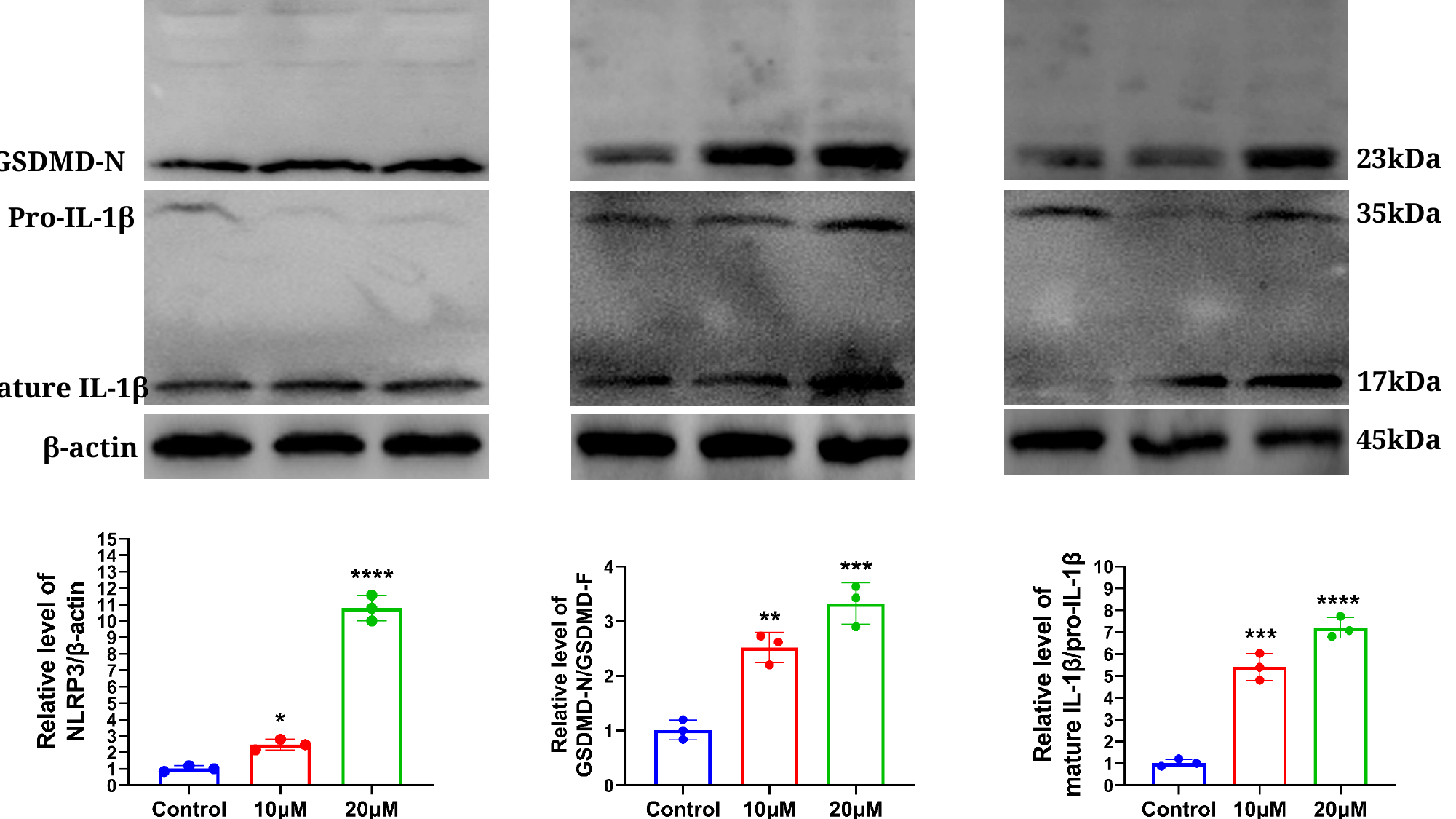

3#
1#
2#
10
10
10
0
0
0
20
20
20
MLD(μM)
110kDa
NLRP3
53kDa
GSDMD-F
23kDa
GSDMD-N
35kDa
Pro-IL-1β
17kDa
mature IL-1β
45kDa
β-actin
Figure 4C

## Slide 3
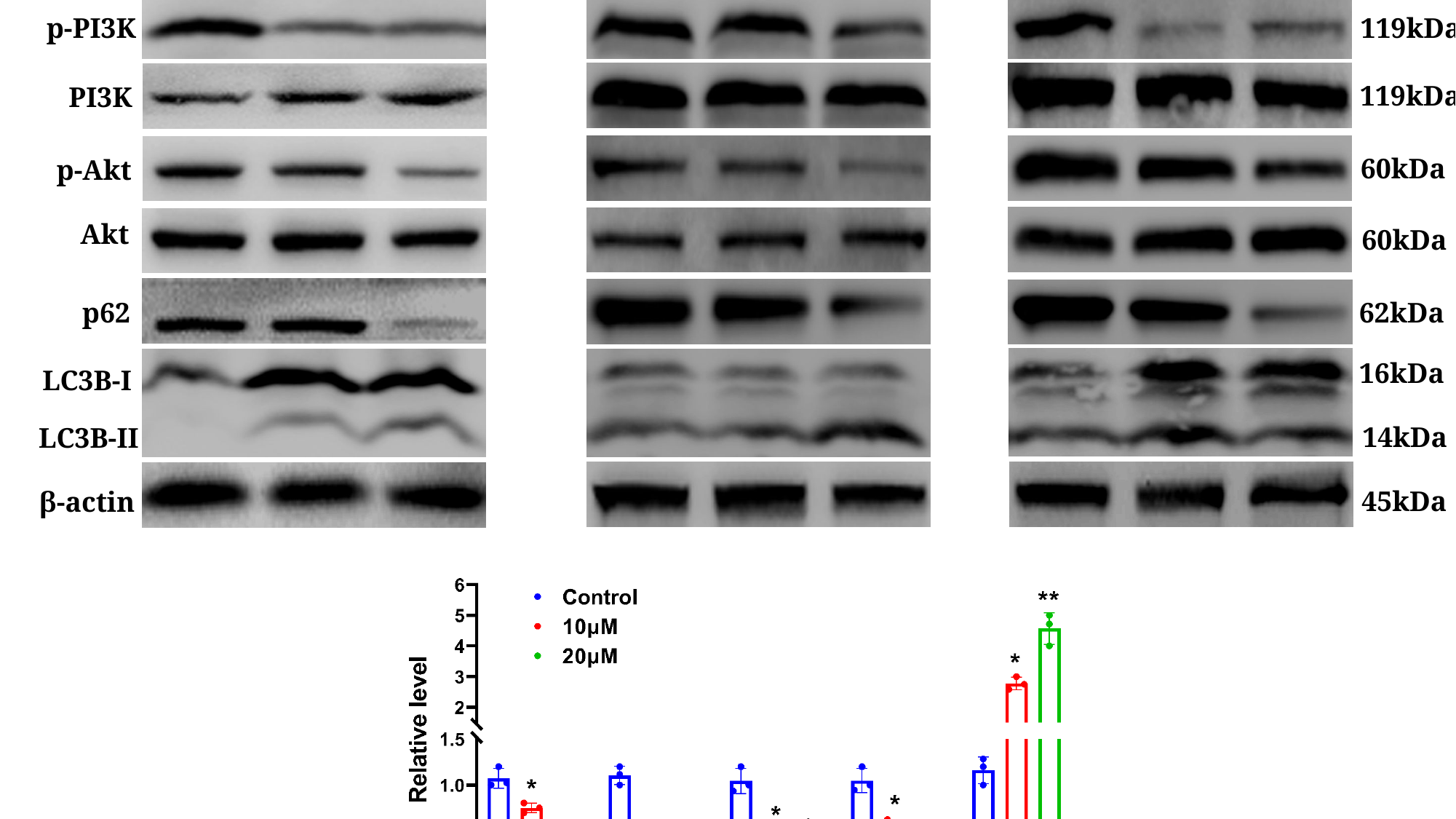

3#
2#
1#
10
10
10
0
0
0
20
20
20
MLD(μM)
289kDa
P-mTOR(S2448)
mTOR
289kDa
119kDa
p-PI3K
119kDa
PI3K
60kDa
p-Akt
Akt
60kDa
p62
62kDa
16kDa
LC3B-I
14kDa
LC3B-II
45kDa
β-actin
Figure 5E

## Slide 4
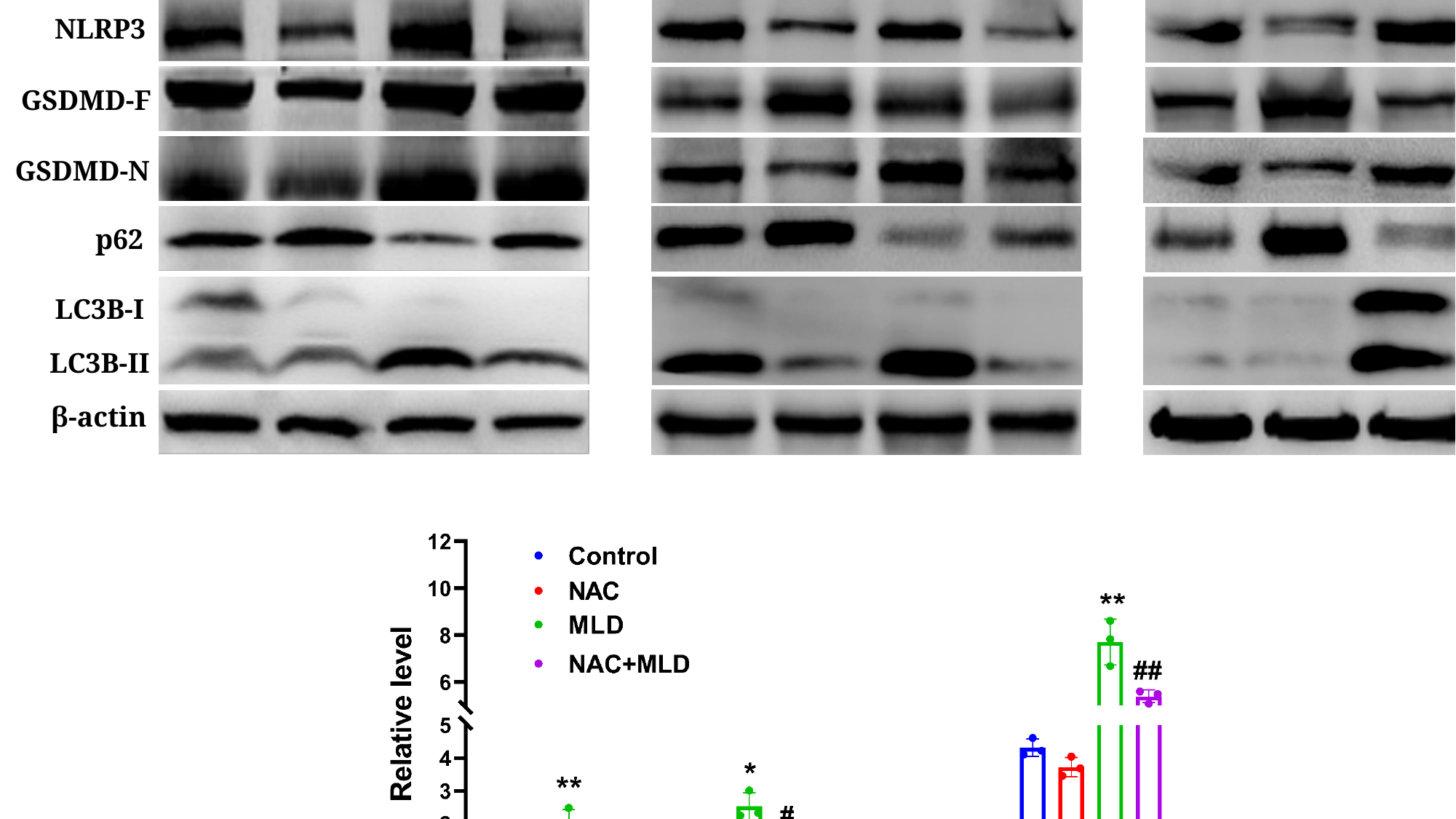

1#
2#
3#
－
－
＋
＋
MLD
－
－
＋
＋
－
－
＋
＋
NAC
＋
－
－
＋
＋
＋
－
－
－
－
＋
＋
NLRP3
110kDa
GSDMD-F
53kDa
23kDa
GSDMD-N
62kDa
p62
16kDa
LC3B-I
14kDa
LC3B-II
β-actin
45kDa
Figure 5G

## Slide 5
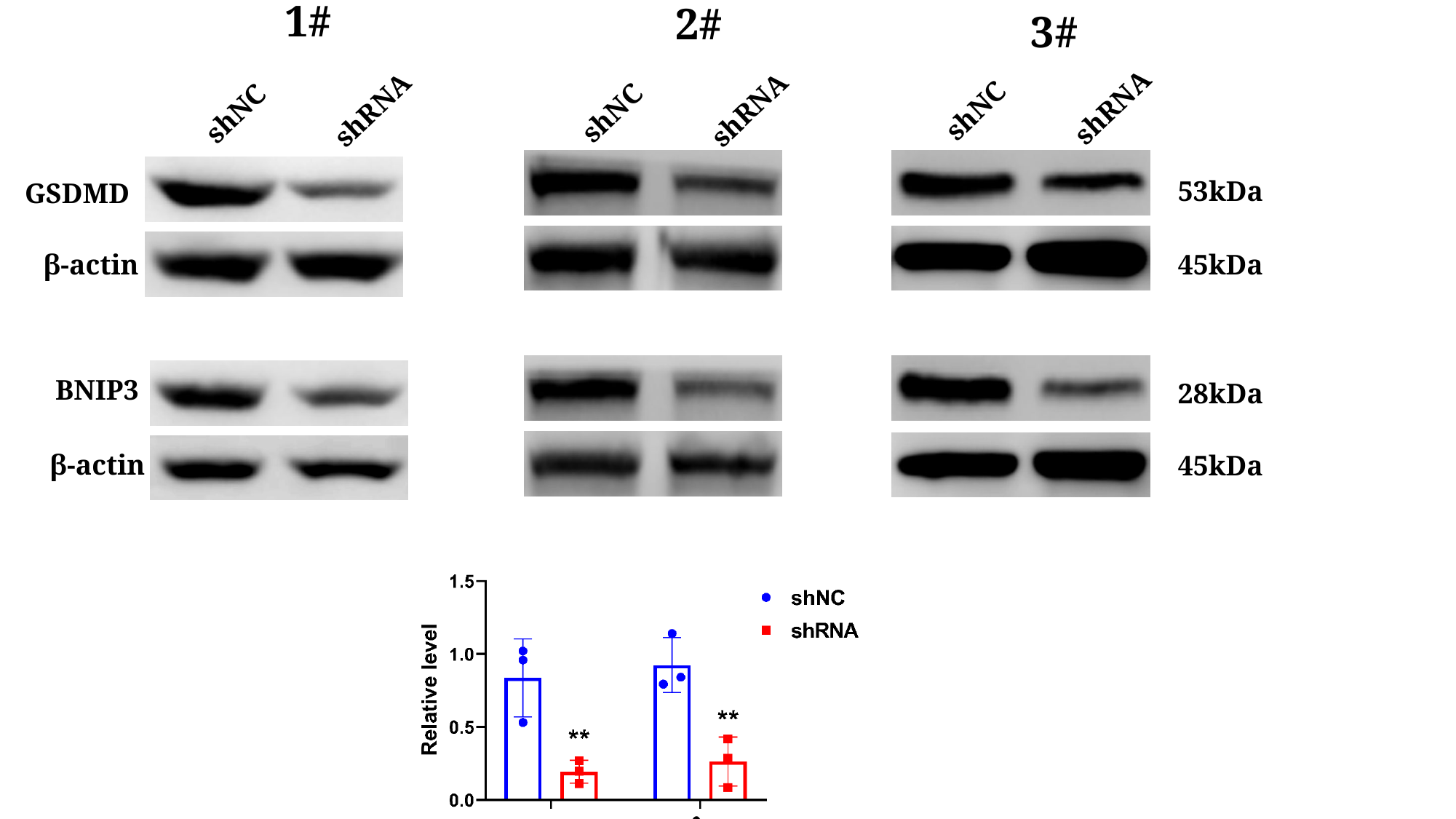

3#
Supplementary figure 5B
1#
2#
shRNA
shRNA
shRNA
shNC
shNC
shNC
53kDa
GSDMD
β-actin
45kDa
BNIP3
28kDa
β-actin
45kDa
BNIP3/β-actin
GSDMD/β-actin
